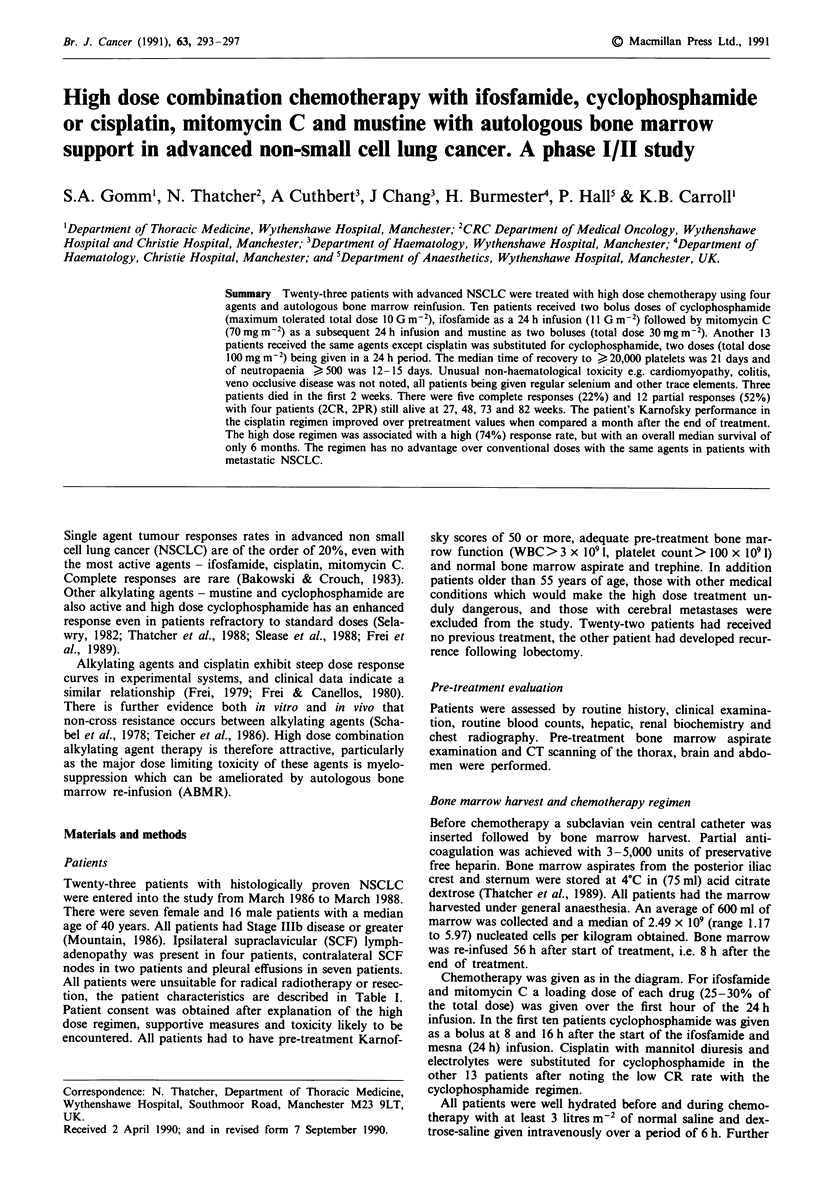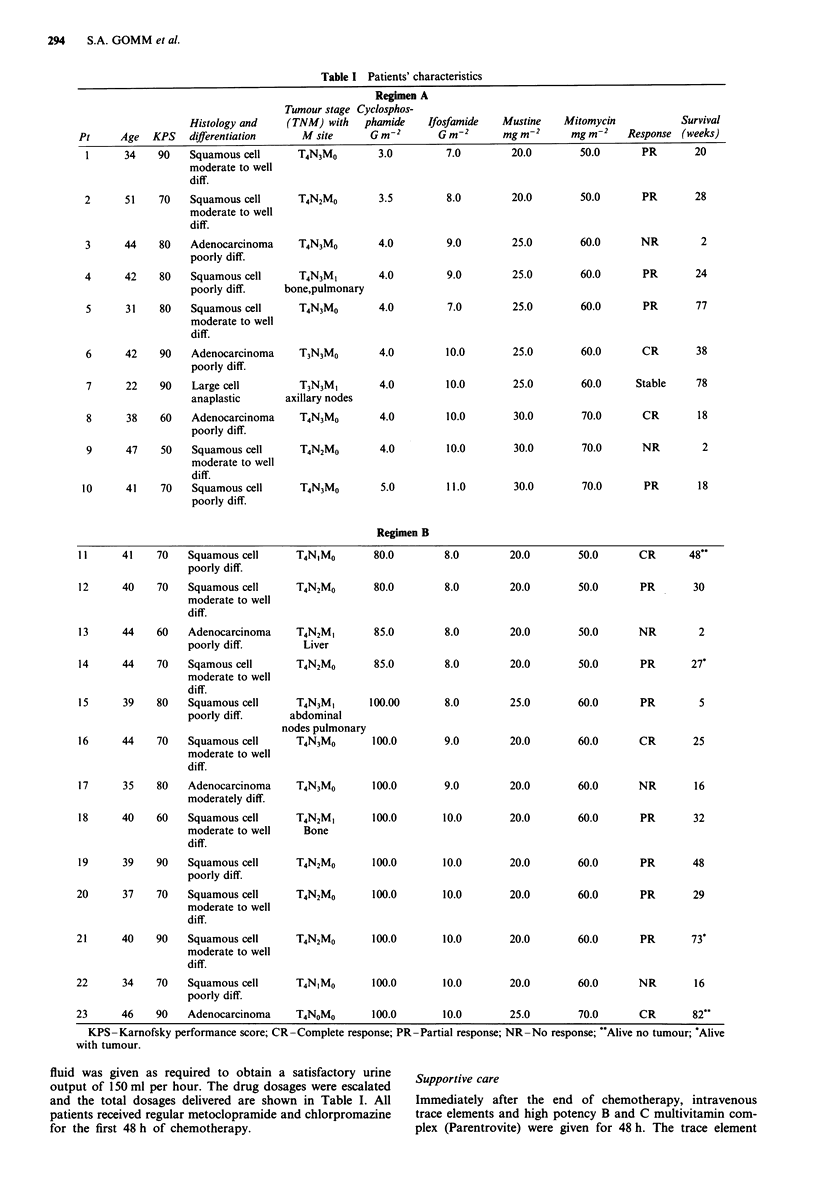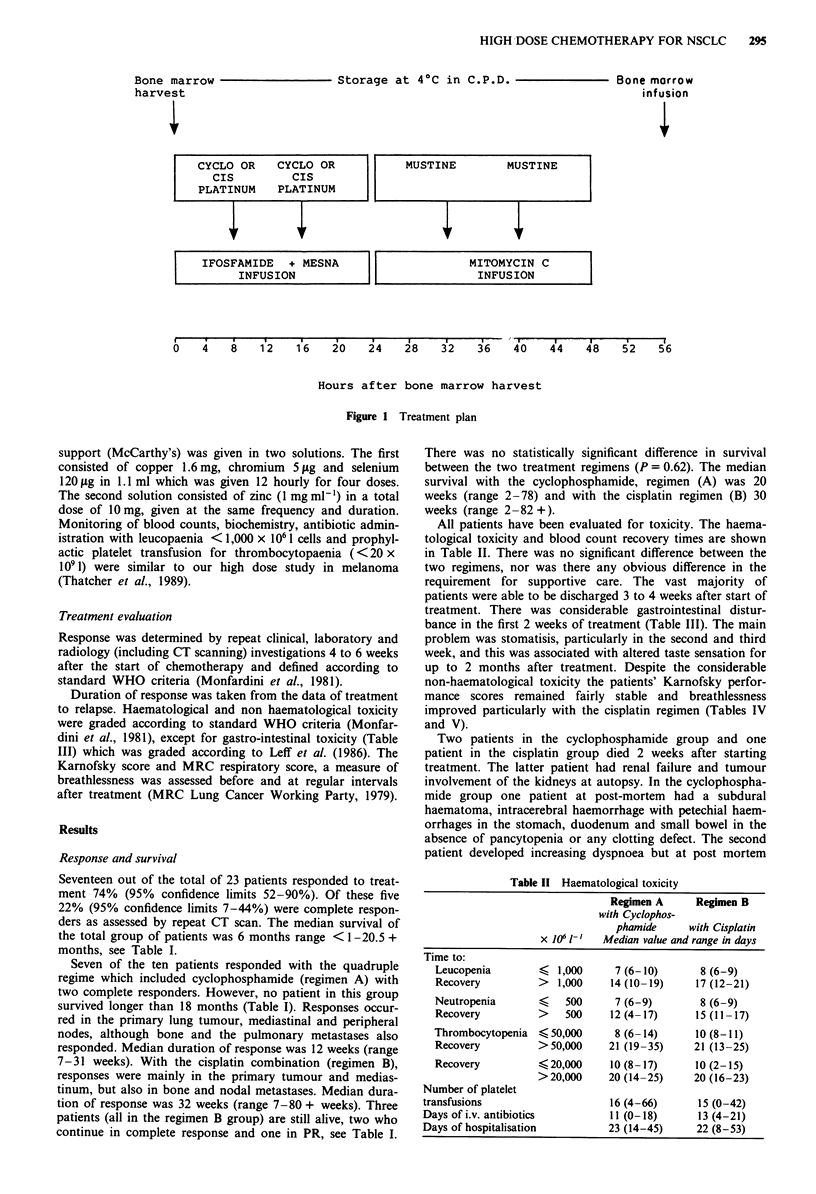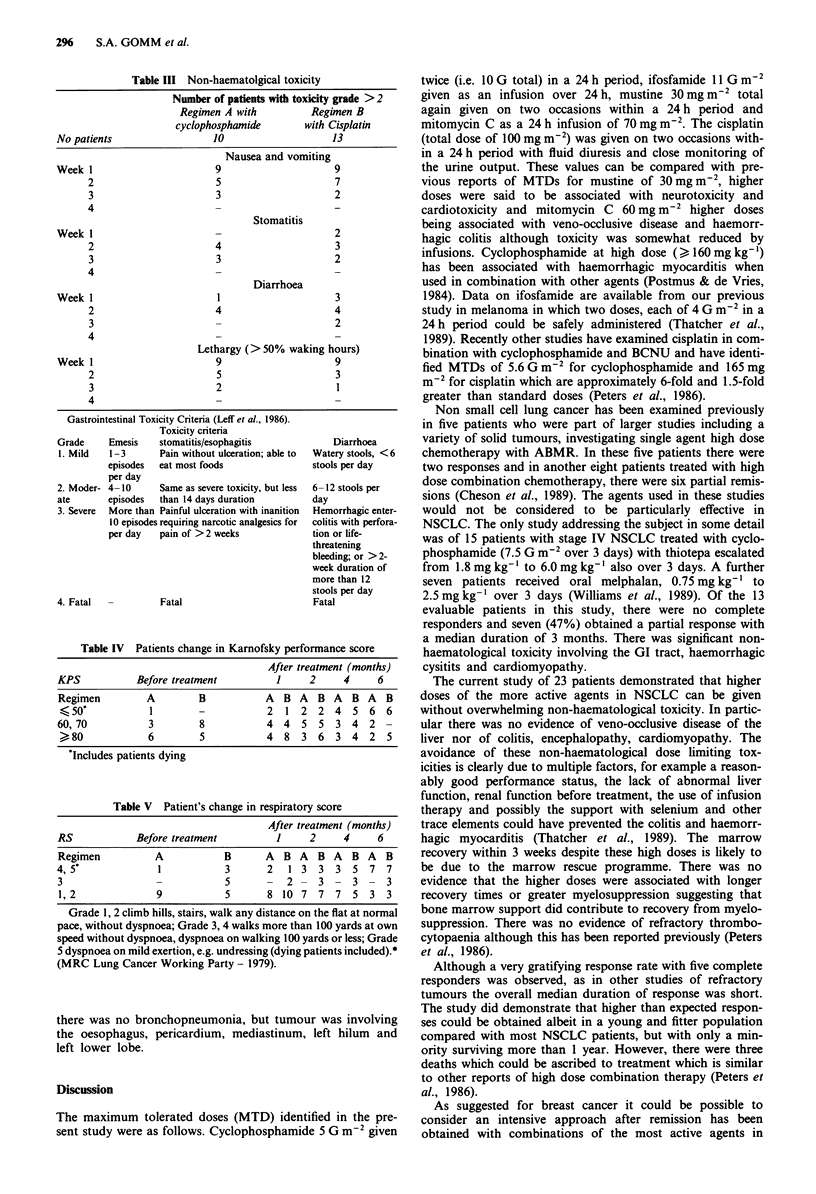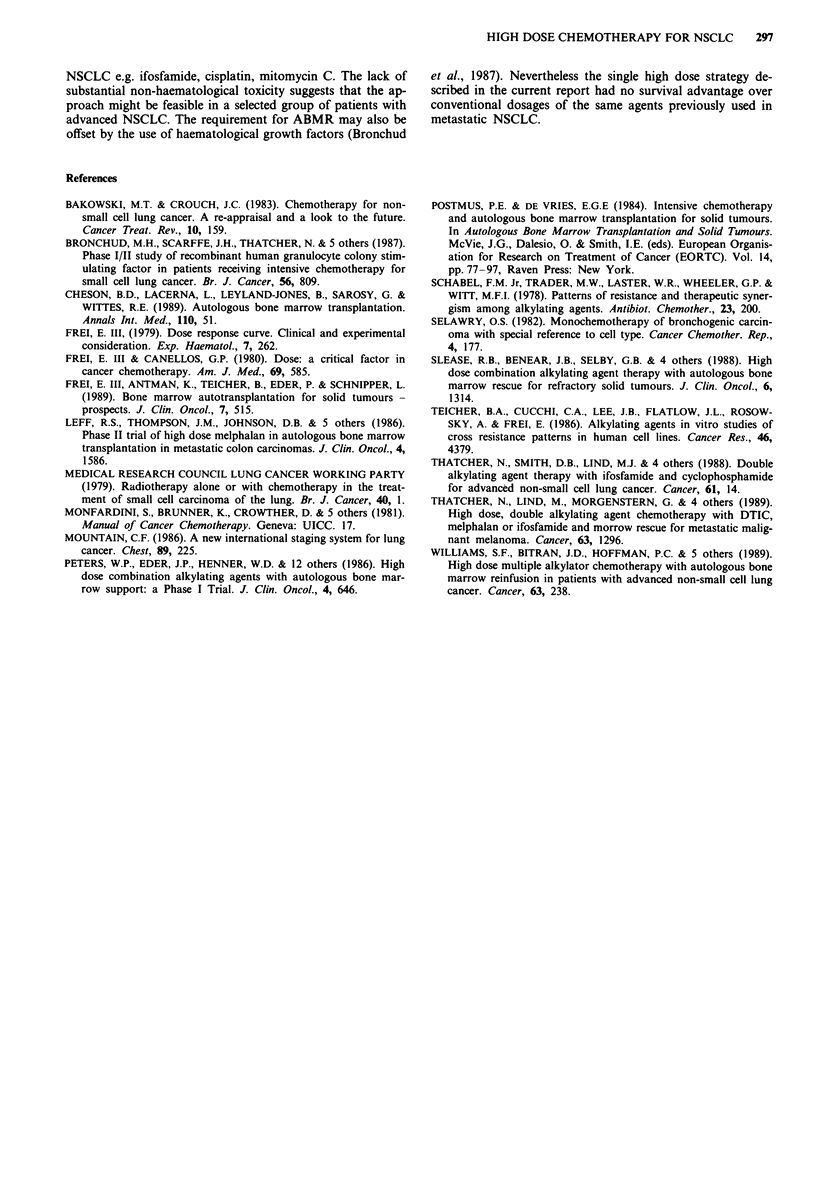# High dose combination chemotherapy with ifosfamide, cyclophosphamide or cisplatin, mitomycin C and mustine with autologous bone marrow support in advanced non-small cell lung cancer. A phase I/II study.

**DOI:** 10.1038/bjc.1991.68

**Published:** 1991-02

**Authors:** S. A. Gomm, N. Thatcher, A. Cuthbert, J. Chang, H. Burmester, P. Hall, K. B. Carroll

**Affiliations:** Department of Thoracic Medicine, Wythenshawe Hospital, Manchester, UK.

## Abstract

Twenty-three patients with advanced NSCLC were treated with high dose chemotherapy using four agents and autologous bone marrow reinfusion. Ten patients received two bolus doses of cyclophosphamide (maximum tolerated total dose 10 G m-2), ifosfamide as a 24 h infusion (11 G m-2) followed by mitomycin C (70 mg m-2) as a subsequent 24 h infusion and mustine as two boluses (total dose 30 mg m-2). Another 13 patients received the same agents except cisplatin was substituted for cyclophosphamide, two doses (total dose 100 mg m-2) being given in a 24 h period. The median time of recovery to greater than or equal to 20,000 platelets was 21 days and of neutropaenia greater than or equal to 500 was 12-15 days. Unusual non-haematological toxicity e.g. cardiomyopathy, colitis, veno occlusive disease was not noted, all patients being given regular selenium and other trace elements. Three patients died in the first 2 weeks. There were five complete responses (22%) and 12 partial responses (52%) with four patients (2CR, 2PR) still alive at 27, 48, 73 and 82 weeks. The patient's Karnofsky performance in the cisplatin regimen improved over pretreatment values when compared a month after the end of treatment. The high dose regimen was associated with a high (74%) response rate, but with an overall median survival of only 6 months. The regimen has no advantage over conventional doses with the same agents in patients with metastatic NSCLC.


Br. J. Cancer (1991), 63, 293-297 © Macmillan Press Ltd., 1991

### High dose combination chemotherapy with ifosfamide, cyclophosphamide or cisplatin, mitomycin C and mustine with autologous bone marrow support in advanced non-small cell lung cancer. A phase I/II study

S.A. Gomm[1], N. Thatcher[2], A Cuthbert[3], J Chang[3], H. Burmester[4], P. Hall[5] & K.B. Carroll[1]

[1]*Department of Thoracic Medicine, Wythenshawe Hospital, Manchester;* [2]*CRC Department of Medical Oncology, Wythenshawe Hospital and Christie Hospital, Manchester;* [3]*Department of Haematology, Wythenshawe Hospital, Manchester;* [4]*Department of Haematology, Christie Hospital, Manchester; and* [5]*Department of Anaesthetics, Wythenshawe Hospital, Manchester, UK.*

**Summary** Twenty-three patients with advanced NSCLC were treated with high dose chemotherapy using four agents and autologous bone marrow reinfusion. Ten patients received two bolus doses of cyclophosphamide (maximum tolerated total dose 10 G $m^{-2}$), ifosfamide as a 24 h infusion (11 G $m^{-2}$) followed by mitomycin C (70 mg $m^{-2}$) as a subsequent 24 h infusion and mustine as two boluses (total dose 30 mg $m^{-2}$). Another 13 patients received the same agents except cisplatin was substituted for cyclophosphamide, two doses (total dose 100 mg $m^{-2}$) being given in a 24 h period. The median time of recovery to $\geqslant$20,000 platelets was 21 days and of neutropaenia $\geqslant$500 was 12-15 days. Unusual non-haematological toxicity e.g. cardiomyopathy, colitis, veno occlusive disease was not noted, all patients being given regular selenium and other trace elements. Three patients died in the first 2 weeks. There were five complete responses (22%) and 12 partial responses (52%) with four patients (2CR, 2PR) still alive at 27, 48, 73 and 82 weeks. The patient's Karnofsky performance in the cisplatin regimen improved over pretreatment values when compared a month after the end of treatment. The high dose regimen was associated with a high (74%) response rate, but with an overall median survival of only 6 months. The regimen has no advantage over conventional doses with the same agents in patients with metastatic NSCLC.

Single agent tumour responses rates in advanced non small cell lung cancer (NSCLC) are of the order of 20%, even with the most active agents – ifosfamide, cisplatin, mitomycin C. Complete responses are rare (Bakowski & Crouch, 1983). Other alkylating agents – mustine and cyclophosphamide are also active and high dose cyclophosphamide has an enhanced response even in patients refractory to standard doses (Selawry, 1982; Thatcher *et al.*, 1988; Slease *et al.*, 1988; Frei *et al.*, 1989).

Alkylating agents and cisplatin exhibit steep dose response curves in experimental systems, and clinical data indicate a similar relationship (Frei, 1979; Frei & Canellos, 1980). There is further evidence both *in vitro* and *in vivo* that non-cross resistance occurs between alkylating agents (Schabel *et al.*, 1978; Teicher *et al.*, 1986). High dose combination alkylating agent therapy is therefore attractive, particularly as the major dose limiting toxicity of these agents is myelosuppression which can be ameliorated by autologous bone marrow re-infusion (ABMR).

### Materials and methods

### *Patients*

Twenty-three patients with histologically proven NSCLC were entered into the study from March 1986 to March 1988. There were seven female and 16 male patients with a median age of 40 years. All patients had Stage IIIb disease or greater (Mountain, 1986). Ipsilateral supraclavicular (SCF) lymphadenopathy was present in four patients, contralateral SCF nodes in two patients and pleural effusions in seven patients. All patients were unsuitable for radical radiotherapy or resection, the patient characteristics are described in Table I. Patient consent was obtained after explanation of the high dose regimen, supportive measures and toxicity likely to be encountered. All patients had to have pre-treatment Karnofsky scores of 50 or more, adequate pre-treatment bone marrow function (WBC$>3\times10^9$ l, platelet count$>100\times10^9$ l) and normal bone marrow aspirate and trephine. In addition patients older than 55 years of age, those with other medical conditions which would make the high dose treatment unduly dangerous, and those with cerebral metastases were excluded from the study. Twenty-two patients had received no previous treatment, the other patient had developed recurrence following lobectomy.

### *Pre-treatment evaluation*

Patients were assessed by routine history, clinical examination, routine blood counts, hepatic, renal biochemistry and chest radiography. Pre-treatment bone marrow aspirate examination and CT scanning of the thorax, brain and abdomen were performed.

### *Bone marrow harvest and chemotherapy regimen*

Before chemotherapy a subclavian vein central catheter was inserted followed by bone marrow harvest. Partial anticoagulation was achieved with 3-5,000 units of preservative free heparin. Bone marrow aspirates from the posterior iliac crest and sternum were stored at 4°C in (75 ml) acid citrate dextrose (Thatcher *et al.*, 1989). All patients had the marrow harvested under general anaesthesia. An average of 600 ml of marrow was collected and a median of $2.49\times10^9$ (range 1.17 to 5.97) nucleated cells per kilogram obtained. Bone marrow was re-infused 56 h after start of treatment, i.e. 8 h after the end of treatment.

Chemotherapy was given as in the diagram. For ifosfamide and mitomycin C a loading dose of each drug (25-30% of the total dose) was given over the first hour of the 24 h infusion. In the first ten patients cyclophosphamide was given as a bolus at 8 and 16 h after the start of the ifosfamide and mesna (24 h) infusion. Cisplatin with mannitol diuresis and electrolytes were substituted for cyclophosphamide in the other 13 patients after noting the low CR rate with the cyclophosphamide regimen.

All patients were well hydrated before and during chemotherapy with at least 3 litres $m^{-2}$ of normal saline and dextrose-saline given intravenously over a period of 6 h. Further

Correspondence: N. Thatcher, Department of Thoracic Medicine, Wythenshawe Hospital, Southmoor Road, Manchester M23 9LT, UK.
Received 2 April 1990; and in revised form 7 September 1990.

**Table I** Patients' characteristics

| Pt | Age | KPS | *Histology and differentiation* | *Tumour stage (TNM) with M site* | *Cyclophosphamide* $G\,m^{-2}$ | *Ifosfamide* $G\,m^{-2}$ | *Mustine* $mg\,m^{-2}$ | *Mitomycin* $mg\,m^{-2}$ | *Response* | *Survival (weeks)* |
|---|---|---|---|---|---|---|---|---|---|---|
| | | | | | **Regimen A** | | | | | |
| 1 | 34 | 90 | Squamous cell moderate to well diff. | $T_4N_3M_0$ | 3.0 | 7.0 | 20.0 | 50.0 | PR | 20 |
| 2 | 51 | 70 | Squamous cell moderate to well diff. | $T_4N_2M_0$ | 3.5 | 8.0 | 20.0 | 50.0 | PR | 28 |
| 3 | 44 | 80 | Adenocarcinoma poorly diff. | $T_4N_3M_0$ | 4.0 | 9.0 | 25.0 | 60.0 | NR | 2 |
| 4 | 42 | 80 | Squamous cell poorly diff. | $T_4N_3M_1$ bone,pulmonary | 4.0 | 9.0 | 25.0 | 60.0 | PR | 24 |
| 5 | 31 | 80 | Squamous cell moderate to well diff. | $T_4N_3M_0$ | 4.0 | 7.0 | 25.0 | 60.0 | PR | 77 |
| 6 | 42 | 90 | Adenocarcinoma poorly diff. | $T_3N_3M_0$ | 4.0 | 10.0 | 25.0 | 60.0 | CR | 38 |
| 7 | 22 | 90 | Large cell anaplastic | $T_3N_3M_1$ axillary nodes | 4.0 | 10.0 | 25.0 | 60.0 | Stable | 78 |
| 8 | 38 | 60 | Adenocarcinoma poorly diff. | $T_4N_3M_0$ | 4.0 | 10.0 | 30.0 | 70.0 | CR | 18 |
| 9 | 47 | 50 | Squamous cell moderate to well diff. | $T_4N_2M_0$ | 4.0 | 10.0 | 30.0 | 70.0 | NR | 2 |
| 10 | 41 | 70 | Squamous cell poorly diff. | $T_4N_3M_0$ | 5.0 | 11.0 | 30.0 | 70.0 | PR | 18 |
| | | | | | **Regimen B** | | | | | |
| 11 | 41 | 70 | Squamous cell poorly diff. | $T_4N_1M_0$ | 80.0 | 8.0 | 20.0 | 50.0 | CR | 48** |
| 12 | 40 | 70 | Squamous cell moderate to well diff. | $T_4N_2M_0$ | 80.0 | 8.0 | 20.0 | 50.0 | PR | 30 |
| 13 | 44 | 60 | Adenocarcinoma poorly diff. | $T_4N_2M_1$ Liver | 85.0 | 8.0 | 20.0 | 50.0 | NR | 2 |
| 14 | 44 | 70 | Sqamous cell moderate to well diff. | $T_4N_2M_0$ | 85.0 | 8.0 | 20.0 | 50.0 | PR | 27* |
| 15 | 39 | 80 | Squamous cell poorly diff. | $T_4N_3M_1$ abdominal nodes pulmonary | 100.00 | 8.0 | 25.0 | 60.0 | PR | 5 |
| 16 | 44 | 70 | Squamous cell moderate to well diff. | $T_4N_3M_0$ | 100.0 | 9.0 | 20.0 | 60.0 | CR | 25 |
| 17 | 35 | 80 | Adenocarcinoma moderately diff. | $T_4N_3M_0$ | 100.0 | 9.0 | 20.0 | 60.0 | NR | 16 |
| 18 | 40 | 60 | Squamous cell moderate to well diff. | $T_4N_2M_1$ Bone | 100.0 | 10.0 | 20.0 | 60.0 | PR | 32 |
| 19 | 39 | 90 | Squamous cell poorly diff. | $T_4N_2M_0$ | 100.0 | 10.0 | 20.0 | 60.0 | PR | 48 |
| 20 | 37 | 70 | Squamous cell moderate to well diff. | $T_4N_2M_0$ | 100.0 | 10.0 | 20.0 | 60.0 | PR | 29 |
| 21 | 40 | 90 | Squamous cell moderate to well diff. | $T_4N_2M_0$ | 100.0 | 10.0 | 20.0 | 60.0 | PR | 73* |
| 22 | 34 | 70 | Squamous cell poorly diff. | $T_4N_1M_0$ | 100.0 | 10.0 | 20.0 | 60.0 | NR | 16 |
| 23 | 46 | 90 | Adenocarcinoma | $T_4N_0M_0$ | 100.0 | 10.0 | 25.0 | 70.0 | CR | 82** |

KPS–Karnofsky performance score; CR–Complete response; PR–Partial response; NR–No response; **Alive no tumour; *Alive with tumour.

fluid was given as required to obtain a satisfactory urine output of 150 ml per hour. The drug dosages were escalated and the total dosages delivered are shown in Table I. All patients received regular metoclopramide and chlorpromazine for the first 48 h of chemotherapy.

### *Supportive care*

Immediately after the end of chemotherapy, intravenous trace elements and high potency B and C multivitamin complex (Parentrovite) were given for 48 h. The trace element

**Figure 1** Treatment plan support (McCarthy's) was given in two solutions. The first consisted of copper 1.6 mg, chromium 5 μg and selenium 120 μg in 1.1 ml which was given 12 hourly for four doses. The second solution consisted of zinc (1 mg ml$^{-1}$) in a total dose of 10 mg, given at the same frequency and duration. Monitoring of blood counts, biochemistry, antibiotic administration with leucopaenia $<1{,}000 \times 10^6$ l cells and prophylactic platelet transfusion for thrombocytopaenia ($<20 \times 10^9$ l) were similar to our high dose study in melanoma (Thatcher *et al.*, 1989).

*Treatment evaluation*

Response was determined by repeat clinical, laboratory and radiology (including CT scanning) investigations 4 to 6 weeks after the start of chemotherapy and defined according to standard WHO criteria (Monfardini *et al.*, 1981).

Duration of response was taken from the data of treatment to relapse. Haematological and non haematological toxicity were graded according to standard WHO criteria (Monfardini *et al.*, 1981), except for gastro-intestinal toxicity (Table III) which was graded according to Leff *et al.* (1986). The Karnofsky score and MRC respiratory score, a measure of breathlessness was assessed before and at regular intervals after treatment (MRC Lung Cancer Working Party, 1979).

### Results

*Response and survival*

Seventeen out of the total of 23 patients responded to treatment 74% (95% confidence limits 52–90%). Of these five 22% (95% confidence limits 7–44%) were complete responders as assessed by repeat CT scan. The median survival of the total group of patients was 6 months range <1–20.5+ months, see Table I.

Seven of the ten patients responded with the quadruple regime which included cyclophosphamide (regimen A) with two complete responders. However, no patient in this group survived longer than 18 months (Table I). Responses occurred in the primary lung tumour, mediastinal and peripheral nodes, although bone and the pulmonary metastases also responded. Median duration of response was 12 weeks (range 7–31 weeks). With the cisplatin combination (regimen B), responses were mainly in the primary tumour and mediastinum, but also in bone and nodal metastases. Median duration of response was 32 weeks (range 7–80+ weeks). Three patients (all in the regimen B group) are still alive, two who continue in complete response and one in PR, see Table I.

There was no statistically significant difference in survival between the two treatment regimens ($P = 0.62$). The median survival with the cyclophosphamide, regimen (A) was 20 weeks (range 2–78) and with the cisplatin regimen (B) 30 weeks (range 2–82+).

All patients have been evaluated for toxicity. The haematological toxicity and blood count recovery times are shown in Table II. There was no significant difference between the two regimens, nor was there any obvious difference in the requirement for supportive care. The vast majority of patients were able to be discharged 3 to 4 weeks after start of treatment. There was considerable gastrointestinal disturbance in the first 2 weeks of treatment (Table III). The main problem was stomatisis, particularly in the second and third week, and this was associated with altered taste sensation for up to 2 months after treatment. Despite the considerable non-haematological toxicity the patients' Karnofsky performance scores remained fairly stable and breathlessness improved particularly with the cisplatin regimen (Tables IV and V).

Two patients in the cyclophosphamide group and one patient in the cisplatin group died 2 weeks after starting treatment. The latter patient had renal failure and tumour involvement of the kidneys at autopsy. In the cyclophosphamide group one patient at post-mortem had a subdural haematoma, intracerebral haemorrhage with petechial haemorrhages in the stomach, duodenum and small bowel in the absence of pancytopenia or any clotting defect. The second patient developed increasing dyspnoea but at post mortem

**Table II** Haematological toxicity

| | $\times 10^6$ $l^{-1}$ | **Regimen A** *with Cyclophosphamide* | **Regimen B** *with Cisplatin* |
|---|---|---|---|
| | | *Median value and range in days* | |
| Time to: | | | |
| Leucopenia | ⩽ 1,000 | 7 (6–10) | 8 (6–9) |
| Recovery | > 1,000 | 14 (10–19) | 17 (12–21) |
| Neutropenia | ⩽ 500 | 7 (6–9) | 8 (6–9) |
| Recovery | > 500 | 12 (4–17) | 15 (11–17) |
| Thrombocytopenia | ⩽ 50,000 | 8 (6–14) | 10 (8–11) |
| Recovery | > 50,000 | 21 (19–35) | 21 (13–25) |
| Recovery | ⩽ 20,000 | 10 (8–17) | 10 (2–15) |
| | > 20,000 | 20 (14–25) | 20 (16–23) |
| Number of platelet transfusions | | 16 (4–66) | 15 (0–42) |
| Days of i.v. antibiotics | | 11 (0–18) | 13 (4–21) |
| Days of hospitalisation | | 23 (14–45) | 22 (8–53) |

**Table III** Non-haematolgical toxicity

| | Number of patients with toxicity grade > 2 | |
|---|---|---|
| | *Regimen A with cyclophosphamide* | *Regimen B with Cisplatin* |
| *No patients* | *10* | *13* |
| | Nausea and vomiting | |
| Week 1 | 9 | 9 |
| 2 | 5 | 7 |
| 3 | 3 | 2 |
| 4 | – | – |
| | Stomatitis | |
| Week 1 | – | 2 |
| 2 | 4 | 3 |
| 3 | 3 | 2 |
| 4 | – | – |
| | Diarrhoea | |
| Week 1 | 1 | 3 |
| 2 | 4 | 4 |
| 3 | – | 2 |
| 4 | – | – |
| | Lethargy (> 50% waking hours) | |
| Week 1 | 9 | 9 |
| 2 | 5 | 3 |
| 3 | 2 | 1 |
| 4 | – | – |

Gastrointestinal Toxicity Criteria (Leff *et al.*, 1986).

| Grade | Emesis | Toxicity criteria stomatitis/esophagitis | Diarrhoea |
|---|---|---|---|
| 1. Mild | 1–3 episodes per day | Pain without ulceration; able to eat most foods | Watery stools, < 6 stools per day |
| 2. Moderate | 4–10 episodes | Same as severe toxicity, but less than 14 days duration | 6–12 stools per day |
| 3. Severe | More than 10 episodes per day | Painful ulceration with inanition requiring narcotic analgesics for pain of > 2 weeks | Hemorrhagic entercolitis with perforation or life-threatening bleeding; or > 2-week duration of more than 12 stools per day |
| 4. Fatal | – | Fatal | Fatal |

**Table IV** Patients change in Karnofsky performance score

| *KPS* | *Before treatment* | | *After treatment (months)* | | | | | | | |
|---|---|---|---|---|---|---|---|---|---|---|
| | | | *1* | | *2* | | *4* | | *6* | |
| Regimen | A | B | A | B | A | B | A | B | A | B |
| ⩽ 50* | 1 | – | 2 | 1 | 2 | 2 | 4 | 5 | 6 | 6 |
| 60, 70 | 3 | 8 | 4 | 4 | 5 | 5 | 3 | 4 | 2 | – |
| ⩾ 80 | 6 | 5 | 4 | 8 | 3 | 6 | 3 | 4 | 2 | 5 |

*Includes patients dying

**Table V** Patient's change in respiratory score

| *RS* | *Before treatment* | | *After treatment (months)* | | | | | | | |
|---|---|---|---|---|---|---|---|---|---|---|
| | | | *1* | | *2* | | *4* | | *6* | |
| Regimen | A | B | A | B | A | B | A | B | A | B |
| 4, 5* | 1 | 3 | 2 | 1 | 3 | 3 | 3 | 5 | 7 | 7 |
| 3 | – | 5 | – | 2 | – | 3 | – | 3 | – | 3 |
| 1, 2 | 9 | 5 | 8 | 10 | 7 | 7 | 7 | 5 | 3 | 3 |

Grade 1, 2 climb hills, stairs, walk any distance on the flat at normal pace, without dyspnoea; Grade 3, 4 walks more than 100 yards at own speed without dyspnoea, dyspnoea on walking 100 yards or less; Grade 5 dyspnoea on mild exertion, e.g. undressing (dying patients included).* (MRC Lung Cancer Working Party – 1979).

there was no bronchopneumonia, but tumour was involving the oesophagus, pericardium, mediastinum, left hilum and left lower lobe.

### Discussion

The maximum tolerated doses (MTD) identified in the present study were as follows. Cyclophosphamide 5 G $m^{-2}$ given twice (i.e. 10 G total) in a 24 h period, ifosfamide 11 G $m^{-2}$ given as an infusion over 24 h, mustine 30 mg $m^{-2}$ total again given on two occasions within a 24 h period and mitomycin C as a 24 h infusion of 70 mg $m^{-2}$. The cisplatin (total dose of 100 mg $m^{-2}$) was given on two occasions within a 24 h period with fluid diuresis and close monitoring of the urine output. These values can be compared with previous reports of MTDs for mustine of 30 mg $m^{-2}$, higher doses were said to be associated with neurotoxicity and cardiotoxicity and mitomycin C 60 mg $m^{-2}$ higher doses being associated with veno-occlusive disease and haemorrhagic colitis although toxicity was somewhat reduced by infusions. Cyclophosphamide at high dose (⩾ 160 mg $kg^{-1}$) has been associated with haemorrhagic myocarditis when used in combination with other agents (Postmus & de Vries, 1984). Data on ifosfamide are available from our previous study in melanoma in which two doses, each of 4 G $m^{-2}$ in a 24 h period could be safely administered (Thatcher *et al.*, 1989). Recently other studies have examined cisplatin in combination with cyclophosphamide and BCNU and have identified MTDs of 5.6 G $m^{-2}$ for cyclophosphamide and 165 mg $m^{-2}$ for cisplatin which are approximately 6-fold and 1.5-fold greater than standard doses (Peters *et al.*, 1986).

Non small cell lung cancer has been examined previously in five patients who were part of larger studies including a variety of solid tumours, investigating single agent high dose chemotherapy with ABMR. In these five patients there were two responses and in another eight patients treated with high dose combination chemotherapy, there were six partial remissions (Cheson *et al.*, 1989). The agents used in these studies would not be considered to be particularly effective in NSCLC. The only study addressing the subject in some detail was of 15 patients with stage IV NSCLC treated with cyclophosphamide (7.5 G $m^{-2}$ over 3 days) with thiotepa escalated from 1.8 mg $kg^{-1}$ to 6.0 mg $kg^{-1}$ also over 3 days. A further seven patients received oral melphalan, 0.75 mg $kg^{-1}$ to 2.5 mg $kg^{-1}$ over 3 days (Williams *et al.*, 1989). Of the 13 evaluable patients in this study, there were no complete responders and seven (47%) obtained a partial response with a median duration of 3 months. There was significant non-haematological toxicity involving the GI tract, haemorrhagic cysitits and cardiomyopathy.

The current study of 23 patients demonstrated that higher doses of the more active agents in NSCLC can be given without overwhelming non-haematological toxicity. In particular there was no evidence of veno-occlusive disease of the liver nor of colitis, encephalopathy, cardiomyopathy. The avoidance of these non-haematological dose limiting toxicities is clearly due to multiple factors, for example a reasonably good performance status, the lack of abnormal liver function, renal function before treatment, the use of infusion therapy and possibly the support with selenium and other trace elements could have prevented the colitis and haemorrhagic myocarditis (Thatcher *et al.*, 1989). The marrow recovery within 3 weeks despite these high doses is likely to be due to the marrow rescue programme. There was no evidence that the higher doses were associated with longer recovery times or greater myelosuppression suggesting that bone marrow support did contribute to recovery from myelosuppression. There was no evidence of refractory thrombocytopaenia although this has been reported previously (Peters *et al.*, 1986).

Although a very gratifying response rate with five complete responders was observed, as in other studies of refractory tumours the overall median duration of response was short. The study did demonstrate that higher than expected responses could be obtained albeit in a young and fitter population compared with most NSCLC patients, but with only a minority surviving more than 1 year. However, there were three deaths which could be ascribed to treatment which is similar to other reports of high dose combination therapy (Peters *et al.*, 1986).

As suggested for breast cancer it could be possible to consider an intensive approach after remission has been obtained with combinations of the most active agents in

NSCLC e.g. ifosfamide, cisplatin, mitomycin C. The lack of substantial non-haematological toxicity suggests that the approach might be feasible in a selected group of patients with advanced NSCLC. The requirement for ABMR may also be offset by the use of haematological growth factors (Bronchud *et al.*, 1987). Nevertheless the single high dose strategy described in the current report had no survival advantage over conventional dosages of the same agents previously used in metastatic NSCLC.

### References

BAKOWSKI, M.T. & CROUCH, J.C. (1983). Chemotherapy for non-small cell lung cancer. A re-appraisal and a look to the future. *Cancer Treat. Rev.*, **10**, 159.

BRONCHUD, M.H., SCARFFE, J.H., THATCHER, N. & 5 others (1987). Phase I/II study of recombinant human granulocyte colony stimulating factor in patients receiving intensive chemotherapy for small cell lung cancer. *Br. J. Cancer*, **56**, 809.

CHESON, B.D., LACERNA, L., LEYLAND-JONES, B., SAROSY, G. & WITTES, R.E. (1989). Autologous bone marrow transplantation. *Annals Int. Med.*, **110**, 51.

FREI, E. III, (1979). Dose response curve. Clinical and experimental consideration. *Exp. Haematol.*, **7**, 262.

FREI, E. III & CANELLOS, G.P. (1980). Dose: a critical factor in cancer chemotherapy. *Am. J. Med.*, **69**, 585.

FREI, E. III, ANTMAN, K., TEICHER, B., EDER, P. & SCHNIPPER, L. (1989). Bone marrow autotransplantation for solid tumours – prospects. *J. Clin. Oncol.*, **7**, 515.

LEFF, R.S., THOMPSON, J.M., JOHNSON, D.B. & 5 others (1986). Phase II trial of high dose melphalan in autologous bone marrow transplantation in metastatic colon carcinomas. *J. Clin. Oncol.*, **4**, 1586.

MEDICAL RESEARCH COUNCIL LUNG CANCER WORKING PARTY (1979). Radiotherapy alone or with chemotherapy in the treatment of small cell carcinoma of the lung. *Br. J. Cancer*, **40**, 1.

MONFARDINI, S., BRUNNER, K., CROWTHER, D. & 5 others (1981). *Manual of Cancer Chemotherapy*. Geneva: UICC. 17.

MOUNTAIN, C.F. (1986). A new international staging system for lung cancer. *Chest*, **89**, 225.

PETERS, W.P., EDER, J.P., HENNER, W.D. & 12 others (1986). High dose combination alkylating agents with autologous bone marrow support: a Phase I Trial. *J. Clin. Oncol.*, **4**, 646.

POSTMUS, P.E. & DE VRIES, E.G.E (1984). Intensive chemotherapy and autologous bone marrow transplantation for solid tumours. In *Autologous Bone Marrow Transplantation and Solid Tumours*. McVie, J.G., Dalesio, O. & Smith, I.E. (eds). European Organisation for Research on Treatment of Cancer (EORTC). Vol. 14, pp. 77–97, Raven Press: New York.

SCHABEL, F.M. Jr, TRADER, M.W., LASTER, W.R., WHEELER, G.P. & WITT, M.F.I. (1978). Patterns of resistance and therapeutic synergism among alkylating agents. *Antibiot. Chemother.*, **23**, 200.

SELAWRY, O.S. (1982). Monochemotherapy of bronchogenic carcinoma with special reference to cell type. *Cancer Chemother. Rep.*, **4**, 177.

SLEASE, R.B., BENEAR, J.B., SELBY, G.B. & 4 others (1988). High dose combination alkylating agent therapy with autologous bone marrow rescue for refractory solid tumours. *J. Clin. Oncol.*, **6**, 1314.

TEICHER, B.A., CUCCHI, C.A., LEE, J.B., FLATLOW, J.L., ROSOWSKY, A. & FREI, E. (1986). Alkylating agents in vitro studies of cross resistance patterns in human cell lines. *Cancer Res.*, **46**, 4379.

THATCHER, N., SMITH, D.B., LIND, M.J. & 4 others (1988). Double alkylating agent therapy with ifosfamide and cyclophosphamide for advanced non-small cell lung cancer. *Cancer*, **61**, 14.

THATCHER, N., LIND, M., MORGENSTERN, G. & 4 others (1989). High dose, double alkylating agent chemotherapy with DTIC, melphalan or ifosfamide and morrow rescue for metastatic malignant melanoma. *Cancer*, **63**, 1296.

WILLIAMS, S.F., BITRAN, J.D., HOFFMAN, P.C. & 5 others (1989). High dose multiple alkylator chemotherapy with autologous bone marrow reinfusion in patients with advanced non-small cell lung cancer. *Cancer*, **63**, 238.